# Clozapine modulates retinoid homeostasis in human brain and normalizes serum retinoic acid deficit in patients with schizophrenia

**DOI:** 10.1038/s41380-020-0791-8

**Published:** 2020-06-02

**Authors:** Francesca Regen, Nicoleta-Carmen Cosma, Lisa R. Otto, Vera Clemens, Lana Saksone, Janine Gellrich, Berk Uesekes, Thi Minh Tam Ta, Eric Hahn, Michael Dettling, Isabella Heuser, Julian Hellmann-Regen

**Affiliations:** Charité – Universitätsmedizin Berlin, corporate member of Freie Universität Berlin, Humboldt-Universität zu Berlin, and Berlin Institute of Health, Department of Psychiatry, Campus Benjamin Franklin, Berlin, Germany

**Keywords:** Molecular biology, Diagnostic markers, Schizophrenia

## Abstract

The atypical antipsychotic clozapine is one of the most potent drugs of its class, yet its precise mechanisms of action remain insufficiently understood. Recent evidence points toward the involvement of endogenous retinoic acid (RA) signaling in the pathophysiology of schizophrenia. Here we investigated whether clozapine may modulate RA-signaling. Effects of clozapine on the catabolism of *all-trans* RA (*at*-RA), the biologically most active metabolite of Vitamin A, were assessed in murine and human brain tissue and peripheral blood-derived mononuclear cells (PBMC). In patients with schizophrenia with and without clozapine treatment and matched healthy controls, *at*-RA serum levels and blood mRNA expression of retinoid-related genes in PBMCs were quantified. Clozapine and its metabolites potently inhibited RA catabolism at clinically relevant concentrations. In PBMC-derived microsomes, we found a large interindividual variability of the sensitivity toward the effects of clozapine. Furthermore, *at*-RA and retinol serum levels were significantly lower in patients with schizophrenia compared with matched healthy controls. Patients treated with clozapine exhibited significantly higher *at*-RA serum levels compared with patients treated with other antipsychotics, while retinol levels did not differ between treatment groups. Similarly, in patients without clozapine treatment, mRNA expression of RA-inducible targets CYP26A and STRA6, as well as at-RA/retinol ratio, were significantly reduced. In contrast, clozapine-treated patients did not differ from healthy controls in this regard. Our findings provide the first evidence for altered peripheral retinoid homeostasis in schizophrenia and suggest modulation of RA catabolism as a novel mechanism of action of clozapine, which may be useful in future antipsychotic drug development.

## Introduction

The atypical antipsychotic clozapine remains the first choice in treatment-resistant schizophrenia (SZ) [1–6]. Despite its potential to cause agranulocytosis/granulocytopenia and metabolic side effects, it exhibits extraordinary antipsychotic properties with negligible risk of extrapyramidal side effects [7]. Clozapine is known for its moderate binding affinity to various neurotransmitter receptors while exhibiting a low affinity to the canonical D2 receptor [8]. These direct neurotransmitter-associated effects cannot sufficiently explain the remarkable efficacy of clozapine in treatment-refractory SZ. This implies that other non-neurotransmitter-associated effects may be expected to underlie its outstanding antipsychotic properties [9–12]. Moreover, both clozapine and its two major metabolites, clozapine-N-oxide (CNO) and N-desmethylclozapine (NDC), exhibit neuroprotective and anti-inflammatory properties [13, 14].

Accumulating evidence suggests that disruption of synaptic functions, triggered at different stages during life, constitutes the neurobiological basis of adaptation deficits in the brain circuitry of patients with SZ [15]. These processes are regulated by paracrine retinoid signaling [16–18]. Several lines of evidence directly point toward disturbed retinoid signaling in schizophrenia [19–25]. All-trans retinoic acid (*at*-RA), the most active metabolite of vitamin A is locally produced and tightly regulated in brain tissue through cytochrome P450- (CYP450) mediated degradation into polar metabolites [26]. Retinoid signaling plays pivotal roles during embryonic development and neuronal differentiation [27, 28]. In the adult brain, RA gradients between synthesizing and degrading sites of brain tissue also control several aspects of neuronal plasticity, including long term potentiation and depression (LTP/LTD) [29, 30], neuritogenesis [31], and the process of metaplasticity [16, 17]. Metaplasticity is required for scaling synaptic strength within a neuronal network in a homeostatic manner and appears to be disturbed in SZ [18, 32–34], possibly due to dysregulated RA signaling in these patients [35]. In addition, evidence from hypothesis-free transcriptomic/proteomic analyses and genome-wide association data point toward altered retinoid signaling in the pathogenesis of SZ [21, 23, 36–38]. Finally, dopaminergic pathways are under direct transcriptional control of retinoids [39]. Based on these associations, first retinoid-targeting clinical trials using bexarotene, a retinoid-X-receptor (RXR)-directed retinoid, show promising results in patients with SZ [40–42].

Based on evidence for dysregulated retinoid homeostasis in SZ, we hypothesized that some of the pleiotropic actions of clozapine might be mediated via direct effects on RA homeostasis. We hypothesized that clozapine might modulate RA homeostasis via direct interactions with local brain *at*-RA catabolism, which is highly prevalent in the human brain and the most relevant regulatory step in brain *at*-RA signaling [43–45]. To identify possible effects of clozapine and its metabolites on retinoid homeostasis, we used a previously established methodology to assess tissue-specific catabolism of RA in murine tissues, human postmortem brain tissue and human peripheral blood-derived mononuclear cells (PBMC) in vitro [46–48]. Furthermore, we assessed various RA homeostasis-related parameters in an observatory clinical study of patients with SZ with and without clozapine medication and matched healthy controls.

## Materials, patients, and methods

### Materials

All chemicals were purchased from Sigma-Aldrich (Taufkirchen, Germany) unless otherwise stated.

### Cell culture and tissue preparation

#### Serum and PBMC isolation

For a detailed description of serum and PBMC isolation, please refer to the Supplement S1. In brief, whole blood was collected in the respective vacuum-extraction tubes and prepared according to the manufacturer’s instructions. Aliquots were stored at −80 °C. For PBMC isolation, heparinized blood was extracted by FICOLL™ density gradient centrifugation, following previously published protocols [49].

### Preparation of crude microsomal and synaptosomal fractions for RA metabolism assays

Human postmortem brain tissue from the superior temporal gyrus of five healthy donors was obtained from the Netherlands Brain Bank (NBB, Netherlands Institute for Neuroscience, Amsterdam). All donors gave written informed consent for brain autopsy and for the use of specimens for research purposes. Mouse tissue was derived from C57/BL6, P0-P3, male and female animals. Animals were sacrificed by decapitation before tissue preparation. All experiments including animals were registered and approved by German regulatory authorities (T 0268/15) and approved by a local institutional review board.

CYP450-containing, metabolically active crude microsomal and synaptosomal fractions from brain tissues and isolated PBMCs were prepared according to previously published protocols with minor modifications [48, 50]. For a detailed description of the procedures, please refer to the Supplementary S1. In brief, tissues or cells from healthy donors (Supplementary Table 1) were homogenized and metabolically active fractions were prepared by differential centrifugation steps. Metabolically active crude synaptosomal/microsomal fractions were stored at −80 °C, protein concentrations were determined by the BCA method (Thermo Fisher, USA).

### RA catabolism assay

In vitro assays to quantify RA metabolism were performed as previously published [51, 52]. A more detailed description of the methods is available in Supplementary S1. In brief, samples contained metabolically active enzyme preparations, the test compound (drug) at the desired final concentration, RA (1 µM), NADPH (800 µg/ml) and assay buffer. Heat-inactivated controls were incubated at 95 °C for 15 min. Reactions were allowed to incubate for 60 min at 37 °C and were stopped by the addition of ice-cold methanol. Subsequently, all samples were centrifuged at 21,000 × *g*, 4 °C and subjected to retinoid analysis. All steps were carried out under dim, yellow light. RA metabolic activity was calculated by comparing RA degradation in metabolically active samples with heat-inactivated controls.

### Participants

Healthy donors and SZ patients within the clinical observational study on RA homeostasis in neuropsychiatric disorders (RAHND; ClinicalTrials.gov Identifier: NCT02439099) were included. The local ethics committee approved the study (EA4/002/13). All patients had a clinical DSM-5 diagnosis of SZ more than five years prior to inclusion were treated in our clinic as in- or outpatients and were on a stable medication with clozapine (*N* = 10) or other antipsychotics (*N* = 10; Supplementary Table 2). All participants were matched for age, weight, BMI, and smoking status, including matched healthy controls (*N* = 10; Table 1). Participants arrived at the laboratory between 8 and 12 a.m. after an overnight fast for blood collection. Each participant provided a total volume of 40 ml of peripheral venous blood.

**Table 1:** Participant characteristics.

	Schizophrenic patients		Healthy controls
	Clozapine	Other medication	
	*N* = 10	*N* = 10	*N* = 10
Age (years ± SD)	42.2 ± 11.1	42.5 ± 13.9	42.2 ± 11.3
Male/female (*n*)	5/5	5/5	5/5
BMI ± SD	25.2 ± 5.0	27.3 ± 5.1	25.9 ± 3.9
Current smokers (*n*)	6	6	6
PANSS total score ± SD^a^	72.5 ± 21.2	86.6 ± 18.1	NA
Years of medication^a^	15.3 ± 9.9	4.5 ± 3.9	NA

*BMI* Body Mass Index, *PANSS* Positive and Negative Syndrome Scale, *NA* not applicable.

^a^Significantly different from healthy controls.

### Serum extraction of retinoids

The extraction of retinoids from human sera was performed by a liquid–liquid extraction procedure using the synthetic retinoid acitretin as an internal standard to assess recovery and account for inter- and intra-assay variability. Liquid–liquid extraction was performed by spiking fractions of 1 ml of serum with internal standard (Acitretin) dissolved in DMSO, then adding 1 vol of acidified ethanol containing 3% (v/v) orthophosphoric acid to 1 vol of patient serum. Samples were vortexed for 1 min and 2 vol of hexane was added. Samples were vigorously vortexed for 15 min and centrifuged at 1560 × *g* at 4 °C for 5 min. The supernatant was evaporated to dryness under a gentle stream of Argon. Samples were resuspended in 1 ml of HPLC running buffer.

### High-performance liquid chromatography

High-performance liquid chromatography (HPLC) was performed as previously described [52] and specified in detail in Supplementary S1. In brief, RA isomers and RA degradation products were quantified using an Agilent 1100-series HPLC system equipped with a Supelco Suplex^®^ column (5 µm, 2.1 × 250 mm; for pharmacological assays) or a Phenomenex Synergi RP 4 µm 80 A column (for serum analyses) and a 1260-series diode-array detector with UV detection at 340 nm for *at*-RA detection and 320 nm for retinol (ROL) detection. Peaks were identified by authentic standards and purity was routinely checked by online spectral analysis.

### Real-time PCR

Blood was collected in PAXgene^®^ tubes (PreAnalytiX GmbH) and RNA was extracted following the manufacturer instructions. Total RNA was then reverse transcribed into cDNA using Revert Aid First Strand cDNA Synthesis Kit™ (Thermo Fisher Scientific Inc., MA, USA). All primers were designed and checked for their quality using the Primer-BLAST software [53]. Primer sequences and further details on qPCR analyses are available in the Supplementary S1.

### Statistical analyses

Numerical analyses were performed using GraphPad statistical software version 5.04 (GraphPad Software, La Jolla, USA). For inhibition characteristics of RA catabolism by clozapine, 3- or 4-parameter nonlinear regression analysis was performed using GraphPad Prism. Values for maximum inhibition and half-maximal inhibitory concentrations (IC50) were calculated based on the regression analysis. Differences between groups were investigated by Student’s *t* test, one-way analysis of variance (ANOVA) with Newman–Keuls multiple comparisons test or Kruskal–Wallis test with Dunn’s multiple comparisons test when appropriate. *P* values < 0.05 were considered statistically significant. Values are given as mean ± S.E.M. * *P* < 0.05, ** *P* < 0.01, *** *P* < 0.001.

## Results

### Clozapine blocks RA catabolism in murine whole brain

**Fig. 1:**
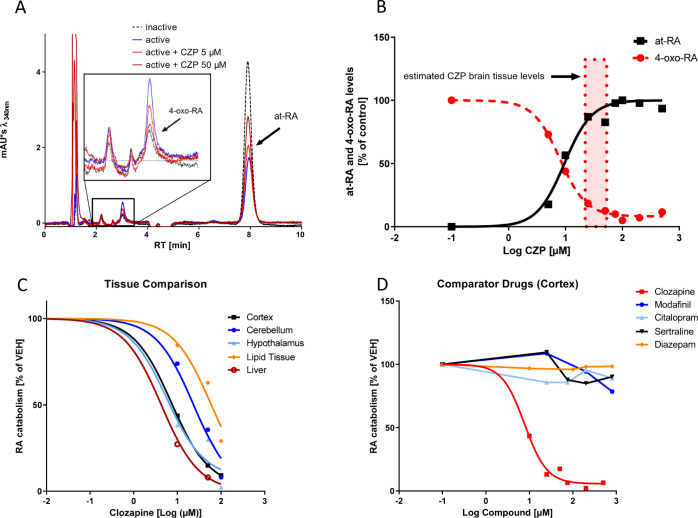
Clozapine blocks RA degradation in murine tissues. **a** HPLC Chromatograms of *at*-RA catabolism assays from representative samples containing heat-inactivated (blue line) or active crude synaptosomal fractions from mouse brain exposed to *at*-RA at 1 µM and treated either with vehicle (black, dashed line) or clozapine at 5 and 50 µM (red lines). **b** Levels of *at*-RA (solid line) and its polar metabolite 4-OXO-RA (dashed line) following in vitro incubation (1 h, 37 °C) with murine brain-derived synaptosomes and *at*-RA 1 µM starting concentration in the presence of various clozapine concentrations. Values are calculated relative to heat-inactivated and vehicle-treated controls. Clozapine significantly inhibits *at*-RA degradation in a dose-dependent manner, blocking on average > 80% of *at*-RA catabolism in microsomal preparations, as calculated by 4-parameter nonlinear regression analysis. The dotted rectangle demonstrates the brain tissue levels of clozapine reached during steady state. **c** *at*-RA catabolism was measured by quantifying the total *at*-RA turnover in clozapine-treated murine tissue samples relative to vehicle-treated samples. Different murine brain regions and other tissues exhibit differential sensitivity toward clozapine. **d** Various other psychotropic drugs were assessed in the same pharmacological assays, including the stimulant modafinil, the two antidepressants citalopram and sertraline and the benzodiazepine diazepam. Neither of the tested drugs exhibited inhibitory effects comparable to clozapine. All assays were performed at least in duplicates using pooled synaptosomes/microsomes from *N* = 3 animals.

In mouse whole brain-derived synaptosomal fractions, clozapine strikingly blocks RA catabolism, starting at concentrations as low as 1 µM when assessed by either reduction of *at*-RA levels or synthesis of the *at*-RA degradation product 4-oxo-RA (Fig. 1a, b).

The representative chromatograms (Fig. 1a) demonstrate low to absent RA catabolism in heat-inactivated samples and substantial RA degradation in active samples by means of reduced *at*-RA peaks and increased RA metabolites. Upon addition of clozapine at 5 or 50 µM, *at*-RA peaks in active synaptosomes increase, while peaks of the RA metabolite 4-oxo-RA decrease in a concentration-dependent manner, indicating that clozapine potently blocks RA catabolism.

Figure 1b further demonstrates the strong inhibitory effect of various clozapine concentrations on RA degradation when measured either by quantification of *at*-RA concentration or by measuring the increase in the concentration of RA metabolites (4-oxo-RA). The half-maximal inhibitory concentrations (IC50 value) for clozapine were 7.9 µM as determined by measuring the reduction in *at*-RA and 7.8 µM as determined by measuring the synthesis of 4-oxo-RA.

Local brain levels of clozapine that are expected to be reached when clozapine is given at therapeutically relevant doses [54] are indicated in the figures by means of rectangles labeled “brain tissue levels”. These levels were calculated based on observations by Wilk et al. and particularly Baldessarini et al., who measured both serum and brain clozapine levels in rats at steady state after intraperitoneal administration [54, 55]. While serum levels in rats reached levels comparable to human serum concentrations, lipophilic clozapine is preferentially distributed to brain tissue resulting on average in 24-fold higher brain concentrations compared with serum levels [54]. In humans, serum clozapine concentrations between 350 and 600 ng/ml are considered to be associated with an optimal therapeutic response [56]. Therefore, brain tissue levels of 8400–14400 ng/ml, which equals 25.7–44.05 µM, are expected.

The increase in *at*-RA and the decrease in the synthesis of 4-oxo-RA in response to clozapine exposure were significantly correlated (Pearson’s *r* = 0.990, *P* < 0.001), suggesting that measuring either the decrease of *at*-RA or the increase of 4-oxo-RA may be adequate to assess RA catabolism.

### Clozapine blocks RA catabolism in various murine tissues

As we were interested in local differences of the effects of clozapine on RA catabolism and because of the known effects of clozapine on energy homeostasis with weight gain as one of the prominent side effects of the drug, we also assessed the impact of clozapine on RA catabolism in murine cerebellum, hypothalamus and in non-neuronal tissues (liver tissue and lipid tissue from visceral fat; Fig. 1c). Clozapine exhibited a differential impact on RA catabolism with most pronounced effects in liver (IC50 = 4.44 µM), cortex (IC50 = 7.30 µM) and hypothalamus (IC50 = 5.76 µM) and less pronounced effects in cerebellum (IC50 = 54.12 µM) and in lipid tissue (IC50 = 699.5 µM).

### Impact of other psychotropic drugs on cerebral RA catabolism

In order to compare the observed effects of clozapine on RA catabolism with other psychotropic drugs, we next tested the effects of the antidepressants citalopram and sertraline, the benzodiazepine diazepam and the stimulant modafinil on RA catabolism, using equally high concentrations, including concentrations above therapeutically relevant tissue levels (see Supplementary Table 3 for estimated brain concentrations) [57–61]. Neither of the tested compounds affected the degradation of RA in mouse cortex-derived synaptosomes to the extent of clozapine (Fig. 1d).

### Effects of clozapine on human tissues

**Fig. 2:**
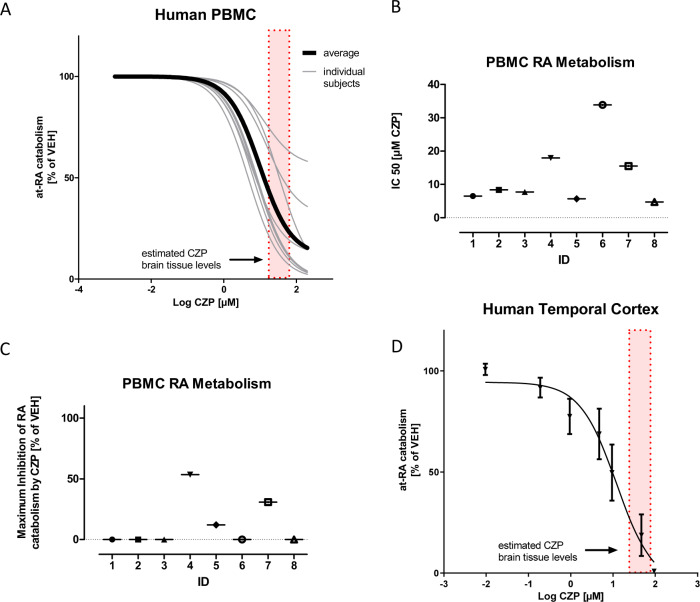
Clozapine blocks *at*-RA degradation in human tissues. **a** Effects of clozapine on *at*-RA degradation in PBMC-derived microsomal preparations were assessed using cells from 8 healthy donors. Clozapine significantly blocked *at*-RA turnover in all subjects (lightweight curves) with an average IC50 value of 9.86 µM (dark bold curve). **b** IC50 values and **c** maximum inhibitability, as calculated by 4-parameter nonlinear regression, exhibited significant variability between the 8 subjects. **d** Clozapine also significantly blocks *at*-RA degradation in pooled human brain-derived synaptosomal preparations derived from postmortem tissue from 5 healthy subjects (IC50: 12.9 ± 1.56 µM). The dotted rectangle demonstrates the brain tissue levels of clozapine during steady state. Values are given as mean and standard error of *n* ≥ 3 experiments.

Next, we analyzed the impact of clozapine on RA catabolism in human PBMC-derived microsomes from eight healthy donors (Fig. 2; for participant characteristics see Supplementary Table 1). Clozapine strikingly inhibited RA catabolism in all samples (Fig. 2a), yet the degree of inhibition varied considerably between subjects. All subjects exhibited IC50 values (mean 12.5 µM, ±9.2) within or even below the estimated brain tissue concentrations of clozapine (Fig. 2a, b). The sensitivity of RA catabolism toward inhibition by clozapine (IC50) also differed markedly between subjects (Fig. 2b). While five out of eight subjects exhibited high sensitivity with IC50 values well below the calculated mean of 12.5 µM, there were three subjects with IC50 values markedly above the mean of 12.5 µM (Fig. 2b). For five out of eight subjects, inhibition was predicted to result in 100% inhibition of RA catabolism (Fig. 2c).

Finally, we assessed RA catabolism in pooled metabolically active synaptosomes from human postmortem brain tissue of five healthy donors (Fig. 2d). Pooled synaptosomes, all prepared from the superior temporal gyrus of the donors, exhibited strong RA catabolic activity. *At*-RA degradation was also strikingly affected by clozapine with an IC50 value of 12.9 µM (Fig. 2d), which is almost identical to the average IC50 value observed for human PBMCs (Fig. 2a) and comparable to murine brain (Fig. 1a).

### Clozapine metabolites N-Desmethylclozapine and Clozapine-N-oxide also affect *at-*RA catabolism

**Fig. 3:**
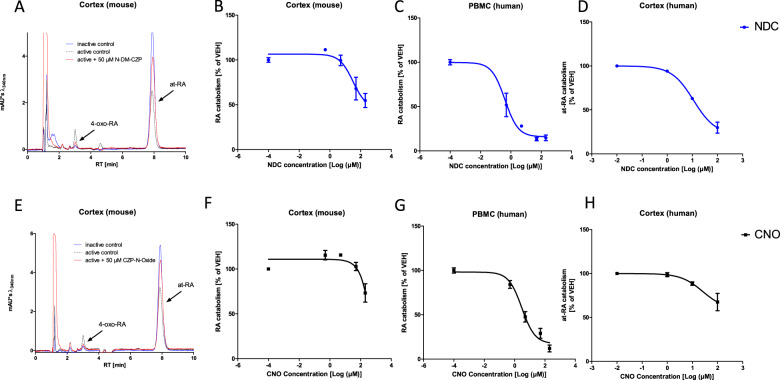
Impact of Clozapine Metabolites on *at*-RA Catabolism. **a** Representative chromatographs of *at*-RA catabolism in murine cortex demonstrate the impact of N-Desmethylclozapine (NDC) on *at*-RA degradation. NDC significantly blocks *at*-RA catabolism in synaptosomes derived from mouse cortex (**b**), human PBMCs (**c**) and human cortex (**d**). Clozapine-n-oxide (CNO) also exhibits significant, yet less pronounced inhibition in murine cortex (**e**, **f**), human PBMCs (**g**) and human cortex (**h**) with IC50 values within the expected brain tissue levels according to 4-paramter nonlinear regression analysis. All experiments were performed at least in duplicates and values are given as mean and standard error.

The two major metabolites, “pharmacologically inert” CNO and NDC, both exhibit neuroprotective and anti-inflammatory properties via unknown mechanisms, starting at concentrations as low as 0.01 µM for CNO and 1 µM for NDC [13]. Interestingly, both metabolites exhibit RA catabolism-blocking properties in murine cortex, pooled human PBMCs and human cortex (Fig. 3). Significant inhibition occurs at low micromolar concentrations that are expected to be reached in clinical practice. The strongest inhibition of RA metabolism was seen for both NDC (IC50 = 0.40 µM) and CNO (IC50 = 2.9 µM) in pooled human PBMC-derived microsomes (Fig. 3c, g).

### Effects of clozapine on serum *at*-RA levels and PBMC mRNA transcripts of RA-related genes in patients with schizophrenia

To assess clinical relevance for the observed effects of clozapine on RA catabolism, we measured for the first time *at*-RA concentration in the serum of patients with SZ who had been on clozapine medication for more than five years (clozapine group), matched patients with SZ taking antipsychotics others than clozapine (no-clozapine group, Supplementary Table 2), and matched healthy controls (Table 1).

**Fig. 4:**
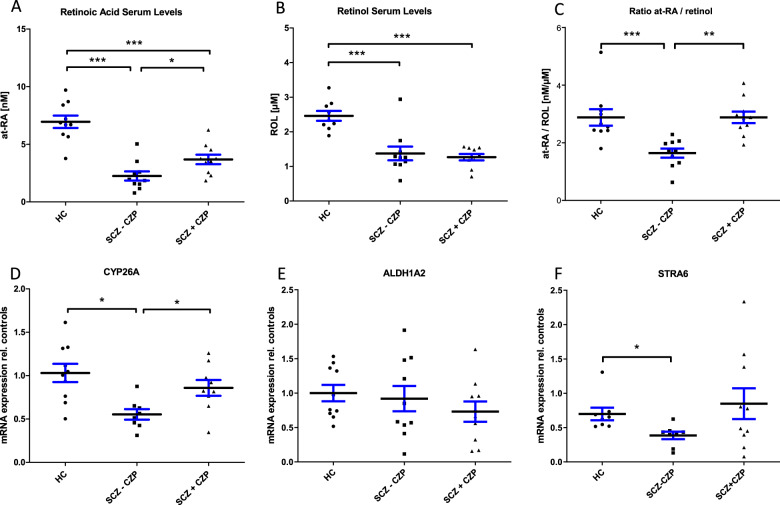
Retinoid Homeostasis in Patients with Schizophrenia with and without Clozapine. **a** Retinoic acid serum levels were measured in healthy controls (HC; *N* = 10), patients with schizophrenia receiving antipsychotic medication other than clozapine (SCZ − CZP; *N* = 10) and patients with schizophrenia currently receiving clozapine (SCZ + CZP; *N* = 10). Serum *at*-RA levels were significantly reduced in patients with schizophrenia compared with controls regardless of medication (***P < 0.0001, one-way ANOVA with Newman–Keul’s post-hoc test). *at*-RA levels in clozapine-treated patients were significantly increased compared with no-clozapine patients (*P < 0.05, one-way ANOVA with Newman–Keul’s post-hoc test). **b** Serum retinol (ROL) levels were significantly reduced in patients with schizophrenia from either medication group compared with HC (***P < 0.0001). **c** The *at*-RA to ROL ratio was significantly reduced only in the SCZ – CZP group. **d** Whole blood mRNA expression of *at*-RA-catabolizing and *at*-RA-inducible CYP26A was also strikingly lowered only in SCZ – CZP subjects while there were no differences between HC and SCZ + CZP (*P < 0.05, one-way ANOVA with Newman–Keul’s post-hoc test). **e**, **f** mRNA levels of the RA-synthesizing enzyme ALDH1A2 did not differ between groups, while mRNA levels of RA-inducible gene STRA6 were significantly decreased in SCZ – CZP compared with healthy controls (*P < 0.05, one-way ANOVA with Newman–Keul’s post-hoc test).

We found significantly reduced serum levels of *at-*RA in patients with SZ compared with matched healthy controls (*P* < 0.0001; Fig. 4a). In patients with SZ, *at-*RA serum levels were lowest in the no-clozapine group of patients with SZ (2.2 ± 1.2 nM) and significantly higher in the clozapine group (3.7 ± 1.3 nM; *P* < 0.05). Both treatment groups exhibited lower *at*-RA levels when compared with healthy controls (7.1 ± 1.8 nM; *P* < 0.001).

ROL levels were also significantly reduced in patients with SZ of the no-clozapine group (1.37 ± 0.19 µM, *P* < 0.001) and the clozapine group (1.27 ± 0.09 µM, *P* < 0.001) compared with healthy controls (2.46 ± 0.43), yet without the effect of medication (Fig. 4b).

Finally, the ratio of *at*-RA to ROL was calculated for each subject (Fig. 4c). Interestingly, ratios were significantly reduced only in the no-clozapine group (1.64 ± 0.16 nM/µM) when compared with healthy controls (2.88 ± 0.28 nM/µM; *P* < 0.001) and subjects from the clozapine group (2.88 ± 0.20 nM/µM; *P* < 0.001). Ratios in the clozapine group did not differ from healthy controls.

PBMC mRNA transcript levels of the RA catabolic enzyme CYP26A (Fig. 4d) and the RA-inducible protein STRA6 (Fig. 4f) both exhibited a similar profile as observed for the *at*-RA/ROL ratio (Fig. 4c). Here, we found significantly lower CYP26A levels in the no-clozapine group of patients with SZ (0.55 ± 0.17) compared with healthy controls (1.0 ±  0.33; *P* < 0.05) or subjects treated with clozapine (0.85 ±  0.27; *P* < 0.05), while there was no difference between clozapine-treated patients and healthy controls. In contrast, there were no main effects of group for the RA-synthesizing enzyme aldehyde dehydrogenase 1A2 (ALDH1A2; Fig. 4e).

**Fig. 5:**
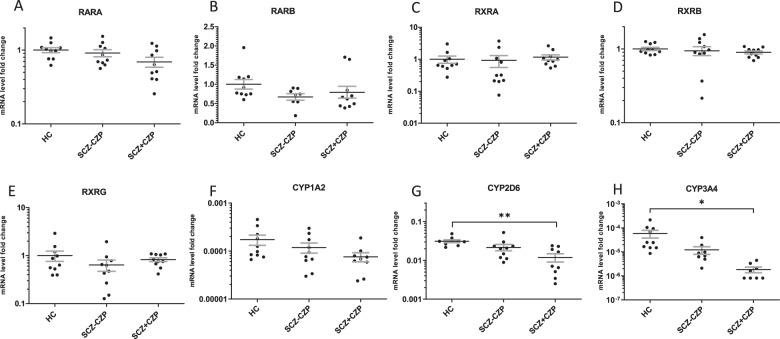
mRNA Expression of RA Receptors and clozapine-metabolizing CYP450 Isozymes. mRNA transcripts of the main retinoid receptors RARA (**a**), RARB (**b**), RXRA (**c**), RXRB (**d**) RXRG (**e**) and clozapine- as well as *at*-RA metabolizing CYP1A2 (**f**), CYP2D6 (**g**) and CYP3A4 (**h**) were detectable in whole blood-derived mRNA. While there were no main effects of group on any of the five receptors (one-way ANOVA), the pattern of RA-inducible RARB is similar to the patterns of STRA6 and CYP26A. Interestingly, both CYP2D6 and CYP3A4 mRNA levels were strikingly decreased in SCZ + CZP patients compared with healthy controls (*P < 0.05, **P < 0.001, one-way ANOVA with Newman–Keul’s post-hoc test).

We also assessed mRNA expression of the retinoic acid receptor (RAR) and RXR α, β and γ isotypes (Fig. 5). While RAR-γ was not detectable, there were no significant main effects of group for any of the receptors. Finally, mRNA expression of non-specific RA-degrading cytochrome isoforms CYP1A2, 2D6, and 3A4 were quantified in PBMCs, exhibiting significantly reduced expression of CYP2D6 and 3A4 in clozapine-treated subjects (Fig. 4f–h).

## Discussion

We demonstrated for the first time that clozapine and its major metabolites specifically inhibit *at-*RA catabolism in a dose-dependent and clinically relevant manner. Furthermore, we provide clinical evidence on significantly reduced retinoid serum levels in patients with schizophrenia and on clozapine positively modulating RA homeostasis in clozapine-treated patients.

Our findings are in line with the “retinoid hypothesis of schizophrenia” [35], which is based on several fundamental findings. First, endogenous retinoids are crucially involved in maintaining neuronal homeostasis by acting as potent endogenous neuroprotective compounds and regulators of inflammation, particularly microglial activation [62, 63]. Moreover, retinoid signaling is increasingly recognized as a key mediator in metaplasticity [16], which is suggested to be disturbed in SZ [34, 64].

In line with the hypothesis of deranged metaplastic processes during the course of illness, several other lines of evidence have also suggested dysregulated retinoid homeostasis in the pathophysiology of schizophrenia [40]. While early studies have clearly identified retinoid dysregulation at the transcriptome level in patients with SZ [37], there is also more recent genome-wide proof for retinoid dysregulation in SZ [21]. Moreover, evidence from retinoid-based treatments supports the hypothesis that specific retinoid-targeting interventions may serve as novel treatment options [41, 42].

When we think about retinoid-based interventions to treat e.g. patients with SZ, it is important to consider that the use of exogenous retinoids may be somewhat compromised by homeostatic adaptations within the locally controlled retinoid signaling network (e.g., induction of local degradation, downregulation of endogenous synthesis) [65]. To overcome this problem, it may be an option to enhance retinoid signaling by means of blocking RA degradation, which is a pivotal step in controlling local RA levels [28, 65, 66]. We previously identified this mechanism for pleiotropic minocycline [46, 48, 67], a tetracycline antibiotic and modulator of microglial activity discussed for use in SZ [68–70]. Out of all antipsychotics available, clozapine is still considered the most effective drug in treatment-refractory SZ. Nevertheless, its precise mechanism of action remains unclear. Based on our findings for minocycline and the fact that catabolism of clozapine involves RA-degrading CYP450 enzymes, we hypothesized clozapine to functionally block endogenous RA catabolism. Further support for this hypothesis comes from a partial overlap of the side effects of clozapine and RA, which include dyslipidemia and impaired bone marrow functions, particularly reduced white blood-cell counts [71, 72].

To assess the effects of clozapine on RA degradation, we functionally measured *at*-RA catabolism in various target tissues in the presence of various clozapine doses. While effects on RA catabolism were expected, the extent to which low, physiological clozapine concentrations blocked *at*-RA degradation was rather unexpected, and so were our findings on NDC and CNO, which equally affect *at*-RA degradation, thereby possibly explaining their pleiotropic effects [13].

Using RA-metabolizing microsomes of donor-derived PBMCs, we provide a potentially valuable tool to quantify a patient-specific response of retinoid homeostasis to clozapine. While our study cannot directly answer the question of whether enhanced retinoid signaling may causally underlie clozapine’s antipsychotic mode of action, our approaches are suitable to correlate the individual retinoid response to clozapine with clinical response in a longitudinal approach, which will be investigated prospectively in future clinical trials.

Retinoid dysregulation in SZ is a convincing theory; however, evidence on functionally altered retinoid signaling in SZ remains associative [19, 21, 25, 35]. We, therefore, assumed alterations in peripheral serum levels of *at*-RA to reflect an overall disturbed retinoid signaling.

Based on our finding that clozapine strikingly affects local *at*-RA degradation we established a three-group design including patients with SZ on an established drug regimen with clozapine (>5 years continuous exposure), patients with SZ taking other antipsychotic drugs and healthy controls, all matched for age, gender, BMI and smoking status. Using a highly sensitive HPLC-based method [50, 73] we were able to identify significantly reduced *at*-RA as well as ROL serum levels, reduced *at*-RA/ROL ratios as well as reduced expression of the RA-inducible and RA-metabolizing CYP26A and the RA-inducible protein stimulated by retinoic acid 6 (STRA6) in schizophrenia patients without clozapine treatment (Fig. 4). Furthermore, confirming our in vitro data, both *at*-RA serum levels, *at*-RA/ROL ratios, and CYP26-levels were increased in patients on clozapine compared with patients on other antipsychotics. Interestingly, PBMC mRNA expression of the RA-synthesizing enzyme ALDH1A2, for which genetic and epigenetic associations with SZ were demonstrated, did not differ between groups [74, 75]. While the expression of RA-inducible RARB, for which associations with schizophrenia have been demonstrated [21, 25] did not differ significantly between groups (Fig. 5b), the tendency toward reduced expression in SCZ-CZP is similar to the pattern observed for CYP26A and STRA6 (Fig. 4). Rather unexpected were findings on reduced CYP2D6 and CYP3A4. On the one hand, reduced expression of non-specific RA-degrading cytochromes may provide a mechanism for increased at-RA serum levels in clozapine-treated subjects. On the other hand, recent in vitro evidence demonstrates the downregulation of CYP2D6 mRNA upon stimulation with retinoids [76], which suggests that reduced levels of CYP2D6 in clozapine patients may also be a consequence of increased RA-signaling. The results must, however, be interpreted with caution, particularly because little is known about the role and functional implications of altered blood-cell-based mRNA expression of CYP450 isozymes.

Limitations of our study include the cross-sectional design and the comparably small sample size of the clinical part. While we were able to show significant inhibition of *at*-RA catabolism at a functional level in various tissues and patient-specific cells, our methodological approach cannot reveal the relative contribution of the different CYP isoforms to the observed effects, which putatively varies between tissues and individual subjects.

While the small sample size was a result of the large effect size of 1 µM clozapine in PBMC-derived microsomes (for details see Supplementary S1), the cross-sectional design cannot provide evidence for a role of clozapine’s RA-modulating effects in its antipsychotic mechanism. Due to methodological limitations, endogenous at-RA metabolites were not detectable in patient-derived samples. Thus, we cannot definitely attribute the alterations to either increased catabolism or decreased anabolism in SZ patients. Despite our careful control for age, weight, and smoking status, confounding effects of eating habits or nutritional preferences on at-RA homeostasis that were not assessed in the present design cannot be definitely ruled out. On the other hand, the large effect sizes that we observed in both our in vitro studies and the patient-specific biological endpoints strongly suggest a clinical relevance of our findings.

Moreover, human brain samples served as generic human brain tissue and were not available from multiple regions. Thus, our present study was not able to resolve the observed effects in a region-specific manner, which should undoubtedly be pursued in future studies. Finally, while our assays were able to detect effects of the test compounds on gross RA catabolism in the respective tissues, we were not able to discriminate the degree to which specific CYP450 isoforms may have contributed to the observed effects. Major challenges are the unspecific effects of pharmacological inhibition [66]. Such an in-depth analysis of which subjects, and via which isozymes, clozapine may specifically block RA catabolism should be the subject of future studies.

In conclusion, we have identified clozapine to strikingly impact RA catabolism in human adult brain tissue and PBMCs, suggesting a retinoid-related mechanism of action of both clozapine and its two major metabolites. Moreover, we identified dysregulation of retinoid homeostasis in patients with SZ at several levels and we revealed potential effects of clozapine on the latter, suggesting normalization of the deficits as part of clozapine’s pleiotropic actions. Our strategy to quantify the impact of clozapine on RA homeostasis parameters in patient-derived material could serve as a versatile and simple tool to assess the patient-specific retinoidergic response to clozapine, which might correlate with later treatment response.

## Supplementary information

Supplemental Material

Supplemental Table 1

Supplemental Table 2

Supplemental Table 3
