# Supplementary material for: Clozapine modulates retinoid homeostasis in human brain and normalizes serum retinoic acid deficit in patients with schizophrenia
Source: Mol Psychiatry. 2020 Jun 2;26(9):5417–28. doi: 10.1038/s41380-020-0791-8 (PMC8589649; doi:10.1038/s41380-020-0791-8)
Supplement: Supplementary file 1 — Supplemental Material [file 41380_2020_791_MOESM1_ESM.pdf]

Supplement S1

The following supplemental information provides further details on the materials used and the methods performed in the present study, particularly on study participants, retinoid analyses, cell culture techniques, qPCR primer sequences and on the preparation of metabolically active synaptosomes / microsomes.

Methods and Materials

Participants

Healthy donors and schizophrenia patients within the clinical observational study on RA homeostasis in neuropsychiatric disorders (RAHND; ClinicalTrials.gov Identifier: NCT02439099) were included. The local ethics committee approved the study (EA4/002/13). All patients had a clinical DSM-V diagnosis of schizophrenia (SZ) more than 5 years prior to inclusion, were treated in our clinic as in- or outpatients and were on a stable medication with clozapine (N = 10) or another antipsychotic (N = 10; supplementary table 2). All participants were matched for age, weight, BMI and smoking-status, including matched healthy controls (N = 10; table 1). Participants arrived at the laboratory between 8 and 12 a.m. after an overnight fast for blood collection. Each participant provided a total volume of 40 ml of peripheral venous blood. In order to estimate the required minimum sample size for the clinical population, the following assumptions were made: Average serum levels of clozapine readily reach > 1 µM under steady state conditions (Hiemke et al., 2018). Thus, effects of 1 µM clozapine on *at*-RA catabolism in human PBMC-derived microsomes was taken as reference to estimate a minimum effect size (Fig. 2 A). Based on the average inhibitory effects of 1 µM clozapine (84.9 % +/- 5.66) vs. vehicle (100 % +/- 10.3) in PBMC-derived microsomes from n = 8 healthy donors, an effect size of d = 1.817 was calculated using the software G*power Version 3.1.9.2. Based on a two-tailed t-test with a power of 95 %, an allocation ratio of 1, and an alpha error probability of 5 %, the required sample size per group was calculated as n = 9.

Cell culture and tissue preparation

Serum and PBMC Isolation

For serum isolation, blood was collected from serum collection tubes (Type SST Advance™, BD Biosciences, Germany). Serum was prepared according to the manufacturer's instructions. Additionally, all samples were either kept in the dark or processed under dim yellow light during the whole procedure. Aliquots were stored at -80 °C. For PBMC isolation, blood was collected from healthy donors (supplementary table 1) using heparinized vacuum-extraction tubes (BD Biosciences) and extracted by FICOLL™ density gradient centrifugation, following previously published protocols (Regen et al., 2017). In brief, samples of heparinized whole blood were carefully layered on top of density medium. After centrifugation, cells were extracted from the interphase, resuspended in PBS, washed twice in PBS and resuspended in RPMI 1640 Medium + GlutaMAX(Biochrom, Germany), containing 25% heat-inactivated fetal calf serum (Biochrom), 1 % Penicillin/Streptomycin (10,000 U / 10 mg per ml; Biochrom, Germany) for cryopreservation.

Tissue Preparation for RA Catabolism Assays

All experiments including animals were registered and approved by German regulatory authorities (T 0268/15). Murine tissues were isolated from newborn C57/BL6-mice (P0-P3) after sacrification by decapitation. Organs were rapidly removed, tissues were prepared and brain regions were dissected using a stereo-microscope (Carl Zeiss, Germany) in ice-cold cell culture media. Samples were weighed and snap-frozen using liquid nitrogen. All samples were stored at -80 °C until further analysis.

Human post-mortem brain tissue from the superior temporal gyrus of 5 healthy donors was obtained from the Netherlands Brain Bank (NBB, Netherlands Institute for Neuroscience, Amsterdam). All donors gave written informed consent for brain autopsy and for the use of specimens for research purposes. All samples and associated data were anonymized to

maintain anonymity of the donors. All procedures on collection and (re-)distribution of the tissue samples were positively evaluated by the Independent Review Board of the VU University (Amsterdam, Netherlands). The experimental procedures on the specimens, including the assessment of retinoid homeostasis, were additionally positively evaluated by the local Charité Ethics Commission. Frozen human brain tissue samples were processed in analogy to murine brain samples.

Preparation of Crude Microsomal and Synaptosomal Fractions

CYP450-containing, metabolically active crude microsomal and synaptosomal fractions from brain tissues and PBMCs were prepared according to previously published protocols with minor modifications (Regen et al., 2016a, Regen et al., 2015). In brief, for preparation of PBMCs, a total of 5 * 10^7 cells were subjected to 50 µl of assay buffer (30mM sodium phosphate buffer, pH 7,4), sonicated for 30 s, centrifuged (10 min. at 1,000 * g at 4 °C), and supernatant was saved. Pellets were resuspended in 50 µl of assay buffer, sonicated and centrifuged again, the resulting supernatants that contained metabolically active, crude synaptosomal / microsomal fractions was then stored at -80 °C while remaining pellets were discarded. For tissue preparations, tissues were resuspended in a 9-fold volume of assay buffer to prepare a 10% homogenate (w/v) by homogenization using a 15 ml glass Teflon homogenizer. Murine tissue preparations were always pooled from three different animals. Following initial centrifugation at 1,000 * g for 10 min. at 4°C, the supernatants were again centrifuged at 100,000 * g for 45 min. at 4°C. The resulting pellets, which contained the metabolically active crude synaptosomal / microsomal fractions were resuspended in assay buffer. Protein concentrations were determined by the BCA method (Thermo Fisher, USA).

RA catabolism assay

In-vitro assays to quantify RA catabolism were performed as previously published (Hellmann-Regen et al., 2015, Regen et al., 2016b). In brief, samples containing metabolically active enzyme preparations were diluted to yield comparable final protein concentrations (100 - 500 µg/ml). Assays were performed in a total reaction volume of 60 µl. Each reaction contained the test compound (drug), *at*-RA (0.1 µM), NADPH (800 µg/ml), metabolically active microsomal / synaptosomal preparations and assay buffer. Reactions were set up by pipetting a range of concentrations of the test drug or vehicle (PBS) to the enzyme-containing samples and allowed to pre-incubate for 30 min. on ice. Heat-inactivated controls were inactivated at 95 °C for 15 min. and also placed on ice. To start the reactions, both NADPH and *at*-RA were added simultaneously, tubes were vortexed and placed in a shaking water-bath incubator for 60 min at 37 °C. After 60 min, reactions were stopped by the addition of 4vol (240 µl) of ice cold methanol. Subsequently, all samples were centrifuged at 21.000 x g, 4° C and subjected to retinoid analysis. All steps were carried out under dim, yellow light. Normalization of *at*-RA metabolic activity was achieved by comparing *at*-RA degradation in metabolically active samples with heat-inactivated controls. Maximum catabolic activity was defined as the reduction in at-RA concentration that occurred over the course of the reaction in metabolically active samples that were treated with vehicle only instead of the compound. Catabolic activity in compound-treated samples was then expressed as a percentage of the maximum catabolic activity. Metabolic activity in heat-inactivated controls was virtually absent and at-RA levels in heat-inactivated controls did not differ over the course of incubation.

at-RA catabolism was initially assessed in whole brain-derived synaptosomal fractions from mouse brains (postnatal day 0-3) in the presence of clozapine at various concentrations covering the clinically relevant range that is reached in murine brain tissue. The samples consisted of pooled synaptosomal fractions from n=3 animals and exhibited strong *at*-RA metabolic activity. In subsequent experiments, various concentrations of clozapine were tested in *at*-RA catabolism assays using pooled murine cortex-derived synaptosomal preparations (n= 3-6 animals).

Serum Extraction of Retinoids

Extraction of retinoids from human sera was performed by a liquid-liquid extraction procedure using the synthetic retinoid acitretin as an internal standard to assess recovery and account for inter- and intra-assay variability. All steps were performed under dim, red light and using glass vials. Liquid-liquid extraction was performed by first spiking fractions of 1 ml of serum with internal standard solved in DMSO to yield a final concentration of 1 µM, then adding 1 vol of acidified ethanol containing 3 % (v/v) orthophosphoric acid to 1 Vol of patient serum. Samples were vortexed for 1 min. and 2 vol of hexane were added. Samples were vigorously vortexted for 15 minutes and centrifuged at 1560 * g at 4 °C for 5 min. The supernatant was completely removed and evaporated to dryness at room temperature under a gentle stream of dry Argon. Samples were subsequently resuspended in 1 ml of HPLC running buffer.

High Performance Liquid Chromatography

High performance liquid chromatography (HPLC) was performed as previously described (Regen et al., 2016b). RA isomers and RA degradation products were quantified using an Agilent 1100-series HPLC system with a binary pump, isocratic elution at a flow rate of 0.65 ml / min and UV detection using a diode array detector (1260-Series) at 340 nm. The system was further equipped with a temperature-controlled column department and a programmable, temperature controlled autosampler. A Supelco Suplex® column (5 µm, 2.1 x 250 mm; Sigma-Aldrich, Taufkirchen, Germany) was used for separation of the cis- and trans- isomers of RA from pharmacological assays and a Phenomenex Synergi RP 4 µm 80A column was used for separation of endogenous retinoids isolated from serum. The mobile phase for the pharmacological assays consisted of acetonitrile, 2% (w/v) ammonium acetate, methanol, glacial acetic acid and n-butanol (69:16:10:3:2; v/v). Elution was performed isocratically for the retinoid quantifications from the pharmacological assays at a flow rate of 0.65 ml/min within a total analysis time of 12 min. For retinoids isolated from serum, a gradient elution was

performed using mobile phase (A) composed of H_2O + 0.1 % formic acid : acetonitrile (15:85 v/v) and a second mobile phase (B) composed of 100 % acetonitrile. Gradient elution was performed (A : B) 0-13 min.: 15:85, 13-14 min.: 0:100, 14-18 min.: 0:100, 18-19 min.: 15:85, runs were terminated after 25 min. All compounds were verified by authentic standards. Peak purity was monitored by online spectral analysis.

Real-Time PCR

Blood was collected in PAXgene® tubes (PreAnalytiX GmbH) and RNA was extracted in accordance with the manufacturer instructions. Total RNA was then reverse transcribed into cDNA using Revert Aid First Strand cDNA Synthesis Kit™ (Thermo Fisher Scientific Inc., MA, USA) and until further measurement all cDNA's were then stored in -20°C. q PCR was performed with LightCycler™ 480 SYBR Green I Master (Roche, Mannheim, Germany) using 500 nM standard primer concentrations, following the recommended instrument protocol from manufacturer's instruction in Applied BiosystemsStepOne™ Real-Time PCR System (CA, USA) to quantify the expression levels. Melting curve analysis was performed to identify specific products with 1.2°C increment from 65 to 95°C. Relative quantity (delta Ct) and melting curve analysis were carried out using the StepOne™ Real-Time PCR System software. All primers were designed and checked for their quality using the Primer-BLAST software (Ye et al., 2012). Sequence (5' > 3') as follows retinoic acid receptor alpha (RARA) (F: ACACTACGAACAACAGCTC, R: TCCACAGTCTTAATGATGCAC), retinoic acid receptor beta (RARB) (F:TGATGGAGTTGGGTGGACTT, R: CGGACTCGCAGTGTAGAAAT), retinoid X receptor alpha (RXRA) (F: GGGCATGAGTTAGTCGCAGA, R: CTGACGGGGTTCATAGGTGA), retinoid X receptor beta (RXRB) (F: CAGAAGCTCAGGCAAACAC, R: TAAGGTCTTTGCGGATGGT), retinoid X receptor G (RXRG) (F: CATGAAGAGGGAAGCTGTG, R: CCACTGGTAGCACATTCTG), aldehyde dehydrogenase 1A1 (ALDH1A2) (F: GTTACAATGCCTTAAATGCCC, R: AGCCAAATTCTCCCATTTCTC), cytochrome P450 26A (CYP26A) (F:

GAAGAGTAAGGGTTTACTTTGC, R: CCCGATGTATTTAAGTTGTTCC), stimulated by retinoic acid receptor-6 (STRA6) (F: AACTGCCGAGACCACACAAC , R: ACGACATTCTCTGGCCCTTC), Cytochrome P450 1A2 (CYP1A2) (F: ATGTGAGCAAGGAGGCTAAGG, R: CATCTCATCGCTACTCTCAGGG), Cytochrome P450 2D6 (CYP2D6) (F: GTGATTCATGAGGTGCAGC, R: GAGTGTCGTTCCCTTAGGG), Cytochrome P450 3A4 (CYP3A4) (F: CACCCCCAGTTAGCACCATTA, R: CCCACGCCAACAGTGATTA). Geometric means of two housekeeping genes, GAPDH and RFLP0, were used for data normalization.

References

HELLMANN-REGEN, J., UHLEMANN, R., REGEN, F., HEUSER, I., OTTE, C., ENDRES, M., GERTZ, K. & KRONENBERG, G. 2015. Direct inhibition of retinoic acid catabolism by fluoxetine. *J Neural Transm (Vienna),* 122**,** 1329-38. doi: 10.1007/s00702-015-1407-3.

HIEMKE, C., BERGEMANN, N., CLEMENT, H. W., CONCA, A., DECKERT, J., DOMSCHKE, K., ECKERMANN, G., EGBERTS, K., GERLACH, M., GREINER, C., GRUNDER, G., HAEN, E., HAVEMANN-REINECKE, U., HEFNER, G., HELMER, R., JANSSEN, G., JAQUENOUD, E., LAUX, G., MESSER, T., MOSSNER, R., MULLER, M. J., PAULZEN, M., PFUHLMANN, B., RIEDERER, P., SARIA, A., SCHOPPEK, B., SCHORETSANITIS, G., SCHWARZ, M., GRACIA, M. S., STEGMANN, B., STEIMER, W., STINGL, J. C., UHR, M., ULRICH, S., UNTERECKER, S., WASCHGLER, R., ZERNIG, G., ZUREK, G. & BAUMANN, P. 2018. Consensus Guidelines for Therapeutic Drug Monitoring in Neuropsychopharmacology: Update 2017. *Pharmacopsychiatry,* 51**,** 9-62. doi: 10.1055/s-0043-116492.

REGEN, F., HERZOG, I., HAHN, E., RUEHL, C., LE BRET, N., DETTLING, M., HEUSER, I. & HELLMANN-REGEN, J. 2017. Clozapine-induced agranulocytosis: Evidence for an immune-mediated mechanism from a patient-specific in-vitro approach. *Toxicol Appl Pharmacol,* 316**,** 10-16. doi: 10.1016/j.taap.2016.12.003.

REGEN, F., HILDEBRAND, M., LE BRET, N., HERZOG, I., HEUSER, I. & HELLMANN-REGEN, J. 2015. Inhibition of retinoic acid catabolism by minocycline: evidence for a novel mode of action? *Exp Dermatol,* 24**,** 473-6. doi: 10.1111/exd.12692.

REGEN, F., LE BRET, N., HILDEBRAND, M., HERZOG, I., HEUSER, I. & HELLMANN-REGEN, J. 2016a. Inhibition of brain retinoic acid catabolism: a mechanism for minocycline's pleiotropic actions? *World J Biol Psychiatry,* 17**,** 634-640. doi: 10.3109/15622975.2015.1036116.

REGEN, F., LE BRET, N., HILDEBRAND, M., HERZOG, I., HEUSER, I. & HELLMANN-REGEN, J. 2016b. Inhibition of brain retinoic acid catabolism: a mechanism for minocycline's pleiotropic actions? *The World Journal of Biological Psychiatry,* 17**,** 634-640.

YE, J., COULOURIS, G., ZARETSKAYA, I., CUTCUTACHE, I., ROZEN, S. & MADDEN, T. L. 2012. Primer-BLAST: a tool to design target-specific primers for polymerase chain reaction. *BMC Bioinformatics,* 13**,** 134. doi: 10.1186/1471-2105-13-134.
