# Supplementary material for: Clozapine modulates retinoid homeostasis in human brain and normalizes serum retinoic acid deficit in patients with schizophrenia
Source: Mol Psychiatry. 2020 Jun 2;26(9):5417–28. doi: 10.1038/s41380-020-0791-8 (PMC8589649; doi:10.1038/s41380-020-0791-8)
Supplement: Supplementary file 2 — Supplemental Table 1 [file 41380_2020_791_MOESM2_ESM.pdf]

Supplementary Table 1. Characteristics of Healthy Donors for PBMS-derived Microsomes

	Healthy Donors<br>N = 8
Age (years ± SD)	33 ± 7.7
Male / Female (n)	3/5
BMI ± SD	20.90 ± 2.16
Current smokers (n)	0

Abbreviations: BMI, Body Mass Index
