# Supplementary material for: Clozapine modulates retinoid homeostasis in human brain and normalizes serum retinoic acid deficit in patients with schizophrenia
Source: Mol Psychiatry. 2020 Jun 2;26(9):5417–28. doi: 10.1038/s41380-020-0791-8 (PMC8589649; doi:10.1038/s41380-020-0791-8)
Supplement: Supplementary file 3 — Supplemental Table 2 [file 41380_2020_791_MOESM3_ESM.pdf]

Supplementary Table 2. Medication

	Clozapine group	Other antipsychotics
Matched Pairs (Nr.)	Clozapine + Comedication (mg per day)	Antipsychotic (mg per day)
1	Clozapine 375, Escitalopram 10	Olanzapine 7.5, Sertraline 100
2	Clozapine 350, Venlafaxin 225, Paliperidone 3	Paliperidone 3, Biperiden 4, Pipamperone 40
3	Clozapine 600, Valproate 2000, Haloperidol 2	Risperidone 3
4	Clozapine 400	Paliperidone 6, Trazodone 50, Duloxetine 60
5	Clozapine 550, Sertraline 75, Lamotrigine 100	Aripiprazole 15, Promethazine 50, Trazodone 50
6	Clozapine 300, Sertraline 100, Pregabalin 200	Aripiprazole 15
7	Clozapine 50	Paliperidone 3
8	Clozapine 350, Risperidone 5	Risperidone 2, Paliperidone 6, Pregabalin 100, Pipamperone 40
9	Clozapine 75, Risperidon 2	Paliperidone 6, Sertraline 100
10	Clozapine 550	Paliperidone 6, Aripiprazole 30, Venlafaxine 262.5
