# Supplementary material for: Clozapine modulates retinoid homeostasis in human brain and normalizes serum retinoic acid deficit in patients with schizophrenia
Source: Mol Psychiatry. 2020 Jun 2;26(9):5417–28. doi: 10.1038/s41380-020-0791-8 (PMC8589649; doi:10.1038/s41380-020-0791-8)
Supplement: Supplementary file 4 — Supplemental Table 3 [file 41380_2020_791_MOESM4_ESM.pdf]

Supplementary Table 3. Local Brain Concentrations

Estimation of Brain Tissue Concentrations

Drug	Serum Levels (Therapeutic Range)	Expected Brain Levels	Reference
**Sertraline**	10 - 150 µg/l	0.72–5.2 mean 2.52 [mg/kg] postmortem	Nedahl et al., 2018 [61]
**Citalopram**	50-110 µg/l	0.052–4.30 mean 1.46 [mg/kg] postmortem	Nedahl et al., 2018 [61]
**Modafinil**	5.88 ± 0.84 µg/ml	not known	Darwish et al., 2009 [63]
**Diazepam**	100 - 2500 µg/l	0.12 [mg/kg] postmortem	Skov et al., 2016 [62]
